# Polarized near-infrared intersubband absorptions in CdSe colloidal quantum wells

**DOI:** 10.1038/s41467-019-12503-z

**Published:** 2019-10-04

**Authors:** Benjamin T. Diroll, Menglu Chen, Igor Coropceanu, Kali R. Williams, Dmitri V. Talapin, Philippe Guyot-Sionnest, Richard D. Schaller

**Affiliations:** 1 Center for Nanoscale Materials, Argonne National Laboratory, 9700S. Cass Avenue, Lemont, IL 60439 USA; 2 Department of Physics, University of Chicago, 2720S. Ellis Avenue, Chicago, IL 60637 USA; 3 Department of Chemistry, University of Chicago, 5735S. Ellis Avenue, Chicago, IL 60637 USA; 4 Department of Chemistry, Northwestern University, 2145 Sheridan Road, Evanston, IL 60208 USA

**Keywords:** Nanoparticles, Optics and photonics, Nonlinear optics

## Abstract

Colloidal quantum wells are two-dimensional materials grown with atomically-precise thickness that dictates their electronic structure. Although intersubband absorption in epitaxial quantum wells is well-known, analogous observations in non-epitaxial two-dimensional materials are sparse. Here we show that CdSe nanoplatelet quantum wells have narrow (30–200 meV), polarized intersubband absorption features when photoexcited or under applied bias, which can be tuned by thickness across the near-infrared (NIR) spectral window (900–1600 nm) inclusive of important telecommunications wavelengths. By examination of the optical absorption and polarization-resolved measurements, the NIR absorptions are assigned to electron intersubband transitions. Under photoexcitation, the intersubband features display hot carrier and Auger recombination effects similar to excitonic absorptions. Sequenced two-color photoexcitation permits the sub-picosecond modulation of the carrier temperature in such colloidal quantum wells. This work suggests that colloidal quantum wells may be promising building blocks for NIR technologies.

## Abstract (web summary)

Multiple infrared lasing and detection technologies exploit intersubband transitions of epitaxial quantum wells, but such transitions are mainly limited to the mid-infrared. Here, the authors report narrow, polarized intersubband transitions up to telecom wavelengths in CdSe colloidal quantum wells.

## Introduction

Colloidal quantum wells (CQWs), often termed nanoplatelets, are a class of colloidal semiconductor materials that, in the case of zinc blende CdSe, are synthesized reliably with atomically precise control of thickness and, consequently, electronic structure^1,2^. CQWs are particularly attractive candidates for light-emitting applications, in which they display nearly thermally limited spectral broadening and polarized emission^2–7^, and as laser gain media, exhibit low-threshold, strong optical gain, and a large gain bandwidth^8–12^. These advantageous properties emerge from the interband transitions of CQWs, but the electronic structure of CQWs also naturally leads to well-defined electronic subbands^13–15^. The discretized electronic structure of epitaxial quantum wells results in narrow intersubband absorption features (between continuous subbands) in photoexcited or doped samples^13,16^. These intersubband absorptions have been exploited extensively in now mature infrared (IR) technologies, such as quantum cascade lasers and IR detectors^17–19^, and they have been proposed for use in ultrafast all-optical switching^20,21^.

Substantial efforts to fabricate thinner epitaxial quantum well structures using wider bandgap semiconductors have extended the energy range of intersubband absorption to the near-IR (NIR)^22–27^, which is particularly coveted for use in telecommunications windows at 1.3 and 1.55 μm wavelengths. However, generation of narrow intersubband absorption resonances in the NIR remains a challenge for epitaxial quantum wells both due to inhomogeneity of the growth process and strain or defects introduced at interfaces. Related to intersubbands observed in quantum wells, intraband absorptions between discreet states have been observed in colloidal^28^ and epitaxial quantum dot systems^29^, but these are in the mid-IR. Recent advances in materials science have created several new classes of materials, which display a quantum well-like electronic structure but do not require epitaxial growth, including van der Waals two-dimensional materials (which have reported mid-IR intersubband absorptions)^30^, quasi-two-dimensional perovskites^31^, and CQWs^2^.

Here, intersubband absorptions are realized in CdSe CQWs of 3.5–6.5 monolayers (MLs are defined as atomic units of Cd and Se; CQWs terminate with cadmium on both large faces making a half layer) spanning the NIR spectral range. By definition CQWs do not require epitaxial growth but they still form atomically sharp interfaces with organic ligand coatings and core/shell heterostructures. Coupled to the synthesis of ensembles with monodisperse thickness^2,9,32^, intersubband absorptions of CQWs are spectrally narrow (30–200 meV) and tunable from 900 to 1600 nm for examined samples. Comparison of photoinduced absorptions and spectroelectrochemical data with static absorption spectroscopy and polarization-resolved measurements confirm that the NIR features are attributable to intersubband absorptions of electrons from the first to second electron subbands. Similar to band-edge absorptions, photo-induced intersubband absorptions in CQWs show multiexcitonic physics and carrier thermalization effects on dynamics and spectra. Last, this work demonstrates that intersubband absorption features can be exploited for sub-picosecond optical modulation.

## Results

### Polarized NIR-induced absorptions of CQWs

**Fig. 1:**
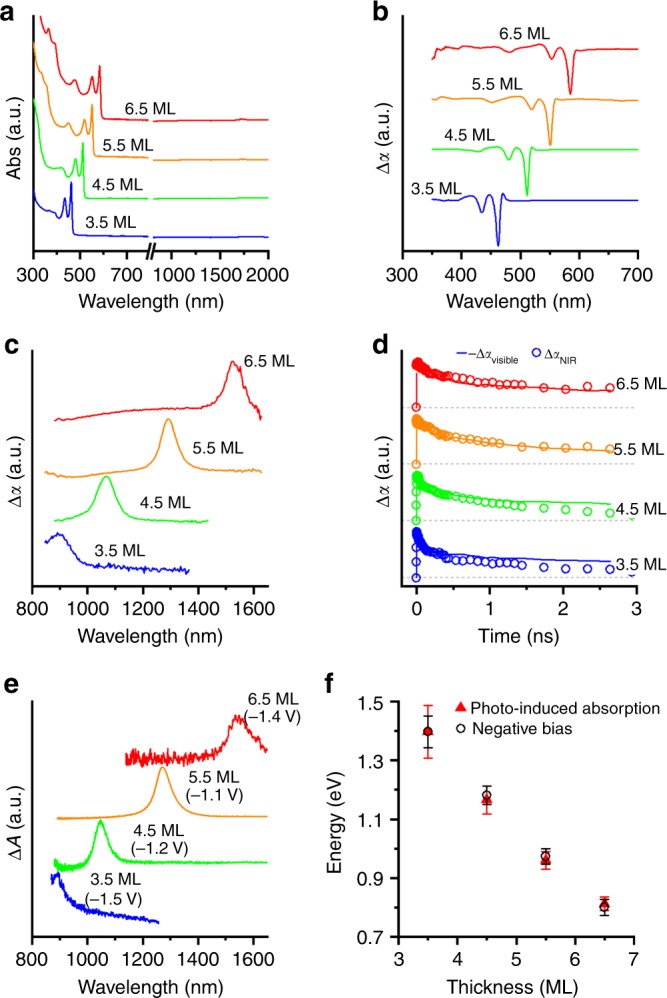
Thickness-dependent static and time-resolved optical properties of CQWs. **a** The 295 K static absorption spectra covering visible and NIR energies of CdSe CQW ensembles with atomically defined thicknesses, as labeled in the panel. Data were collected in two separate measurements for the visible and NIR (merged at 800 nm). **b** Visible- and **c** NIR-probe transient absorption spectra of the same samples with 400 nm excitation at 10 ps delay. **d** Transient absorption dynamics of the first excitonic bleach feature (shown as solid lines) and the NIR-induced absorption feature (shown as open circles) for each of the samples at comparable fluence. **e** Static change in absorption spectra (Δ*A*) of electrochemically pumped CQW samples under applied bias (vs. SCE listed on the plot) in an electrolyte. **f** Fitted energy of the photoinduced (closed red triangles) and electrochemically induced (open black circles) NIR absorption feature vs. the thickness (in monolayers of CdSe units). The error bars represent the full-width at half-maximum of the features

Typical static absorption spectra of CdSe CQWs are shown in Fig. 1a (transmission electron microscopy is shown in Supplementary Fig. 1.) CdSe CQWs are transparent in the NIR and show excitonic absorptions at visible wavelengths determined by their two-dimensional electronic structure. The excitonic wavelengths are diagnostic of the CQW thickness, here shown for 3.5 to 6.5 ML samples. Thinner or thicker CQWs of CdSe^33,34^ or other compositions^2^ (e.g., CdS or CdTe) are anticipated to show similar phenomena to the following, but there is currently less synthetic control over those compositions. Upon absorption of an above-bandgap photon, the CQWs display three bleaching features, shown in Fig. 1b, which represent heavy-hole (HH), light-hole (LH), and spin–orbit (SO) hole transitions to the first electronic shelf (*E*_1_)^2^. At the same time, and displayed in Fig. 1c, spectrally narrow absorption features emerge in the NIR spectral window. Similar NIR-photoinduced absorptions have been reported once before^35^, but in that case they were attributed to an unspecified process, which roughly tracks the energy of the HH transition. In a recent report on the properties of PbSe/CdSe dot-on-plate heterostructures, NIR-photoinduced absorptions are also observed, but not addressed directly^36^. The energetic position of the NIR absorption features depends on the CQW thickness: identical photoinduced absorptions were observed in other CQW samples with the same thickness. The energy of the NIR feature red-shifts for thicker CQWs and particularly noteworthy are the photoinduced absorption features of the 5.5 and 6.5 ML samples, which appear at the 1.3 and 1.55 μm bands used in telecommunications. Based on a comparison of the visible bleach feature with the strength of the NIR absorption, the oscillator strength of the NIR absorptions are approximately one-fortieth (i.e., 10^−15^ to 10^−16^ cm^−2^) of the band-edge absorption resonances, which depend strongly on lateral area^37^.

Two additional experiments support that the NIR-photoinduced absorptions arise from intersubband transitions. First, the kinetics of the visible bleaching feature and the NIR-induced absorption were directly compared for the same samples at similar pump fluence. As shown in Fig. 1d, the kinetic traces of these features overlap for each of the samples measured, which provides evidence that the NIR-photoinduced absorption occurs when excitons are present in the nanostructures. Second, spectroelectrochemical results demonstrate that NIR absorption features are obtained for CQW films when electrochemically charged. Solid thin films of CQWs are prepared by drop-casting followed by soaking for 1 min in a 5 mg mL^−1^ methanolic solution of benzoic acid. Similar to earlier reports on electrochromic quantum dots^38^, negative applied biases lead to filling of the CdSe CQW conduction band with electrons, which reveals intersubband absorption features of electrons (Fig. 1e). The conductivity of the CQW films also increases as electrons fill the conduction band (Supplementary Fig. 2). As plotted in Fig. 1e, the NIR-induced absorption features observed in spectroelectrochemical experiments are nearly identical in energy to those observed after photoexcitation. These experiments indicate that CQWs display intersubband absorptions of electrons in the conduction band with energies dictated by the CQW thickness.

**Fig. 2:**
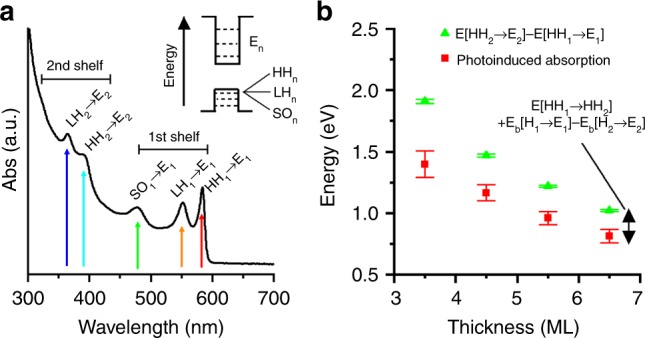
Electronic structure and optical properties of colloidal quantum wells. **a** Absorption spectrum of a 6.5 ML CQW with specific interband absorption resonances labeled according to the inset cartoon band diagram. **b** Thickness-dependent energies, in green circles, of the energy experimental difference between energy ${E}\left[ {{\mathrm{HH}}_2 \to {E}_2} \right]$ and the energy ${E}\left[ {{\mathrm{HH}}_2 \to {E}_1} \right]$ of the *n* = 2 and *n* = 1 interband transitions, determined from optical absorption spectra. Transition energies are estimated based on analysis of the relevant second derivative minima of the absorption spectra, with errors estimated from the spectral bandwidth of the measurement (~1.5 nm). Also plotted in red squares are experimentally observed intersubband absorptions. The error bars of the photoinduced absorption feature reflect the full-width at half-maximum

To assign the specific transition responsible for the NIR-induced absorption feature, the electronic structure of the CQWs is illustrated in Fig. 2a with transitions of a 6.5 ML CQW sample. CQWs’ electronic structure consists of electron and hole states with quantum number subscripted *n*; hole states split into HH, LH, and SO bands. The three lowest-energy static absorption features are identified as HH_1_, LH_1_, and SO_1_ transitions to the *E*_1_ state, respectively^2^. The fourth and fifth transitions observed in the optical spectrum are identified as HH_2_ and LH_2_ band transitions to the second electronic shelf of the conduction band (*E*_2_), consistent with effective mass models of the energetic spacing (Supplementary Fig. 3) and in keeping with the fact that the strongest interband transitions of quantum wells have the same quantum number^33,39^.

An additional factor that complicates the interpretation of the absorption spectrum of CQWs is the exciton binding energy, which reduces the energy of the observed optical resonances with respect to the band positions. Each interband (quantum number *n*) transition of the CQWs has a distinct exciton binding energy, denoted $E_{\mathrm{b}}\left[ {{\mathrm{HH}}_n \to E_n} \right]$ for the HH to electron band transitions. Therefore, the energy differences of electron and hole bands of different quantum numbers are not directly accessible from absorption measurements both due to selection rules of interband transitions (Δ*n* = 0 rule) and exciton binding energy effects. Given the evidence that the NIR-induced absorption feature reflects an intersubband absorption of electrons, and compared with spectroscopic evidence in Fig. 2b, the main interssubband absorption for electrons in a wide-gap material such as CdSe is expected to be for the *E*_1_ → *E*_2_ transition. This transition is allowed, with strong expected polarization, addressed below^40–42^. A more complete derivation may be found in the Supplementary Information, but due to these two adjustments, the energy of the first electron intersubband absorption can be estimated from absorption spectra as

1 $$	E\left[ {E_1 \to E_2} \right] = \left( {E\left[ {{\mathrm{HH}}_2 \to E_2} \right] - E\left[ {{\mathrm{HH}}_1 \to E_1} \right]} \right) \\ 	- \left( {E\left[ {{\mathrm{HH}}_1 \to {\mathrm{HH}}_2} \right] + E_{\mathrm{b}}\left[ {{\mathrm{HH}}_1 \to E_1} \right] - E_{\mathrm{b}}\left[ {{\mathrm{HH}}_2 \to E_2} \right]} \right),$$

which takes into account the energy of optically allowed interband and intersubband transitions, indicated with arrows as, for example, $E\left[ {E_1 \to E_2} \right]$, and the exciton binding energies of interband transitions, indicated as for example, $E_{\mathrm{b}}\left[ {{\mathrm{HH}}_1 \to E_1} \right]$. In this case, because hole intersubband transitions are not observed, and the exciton binding energies of the relevant interband transitions are inexactly defined, the problem is underdetermined. The experimentally observed energy difference between interband transitions denoted $\left( {E\left[ {{\mathrm{HH}}_2 \to E_2} \right] - E\left[ {{\mathrm{HH}}_1 \to E_1} \right]} \right)$ represents an upper limit on the energy of electron intersubband transition. A similar argument applies to the hole intersubband transitions.

Figure 2b shows this empirical upper energy limit and the energy positions of experimentally observed NIR intersubband absorptions, which fall below this empirical upper limit. The gaps is 513, 322, 262, and 209 meV for 3.5, 4.5, 5.5, and 6.5 ML CQWs, respectively. This is consistent with the additional energy $E\left[ {{\mathrm{HH}}_1 \to {\mathrm{HH}}_2} \right]$ necessary for the first HH intersubband transition *and* differences in the exciton binding energy of the respective interband transitions ($E_{\mathrm{b}}\left[ {{\mathrm{HH}}_1 \to E_1} \right] - E_{\mathrm{b}}\left[ {{\mathrm{HH}}_2 \to E_2} \right]$), both of which decrease for thicker CQWs. The precise contribution of the hole transitions and the differences in exciton binding energies is not known. Given available estimates of the exciton binding energy $E_{\mathrm{b}}\left[ {{\mathrm{HH}}_1 \to E_1} \right]$, a lower limit for the higher exciton binding energies may be estimated with a Rydberg model for two-dimensional hydrogenic excitons as $E_{\mathrm{b}}\left( n \right) \propto \left( {n - 1/2} \right)^{ - 2}$, although the accuracy of this model is questioned in other two-dimensional materials^43^. This estimate suggests a reduction in the exciton binding energy from the $E_{\mathrm{b}}\left[ {{\mathrm{HH}}_1 \to E_1} \right]$ to $E_{\mathrm{b}}\left[ {{\mathrm{HH}}_2 \to E_2} \right]$ by roughly a factor of 10, which places the differences of the exciton binding energies of the first and second interband transitions at 90–360 meV.

**Fig. 3:**
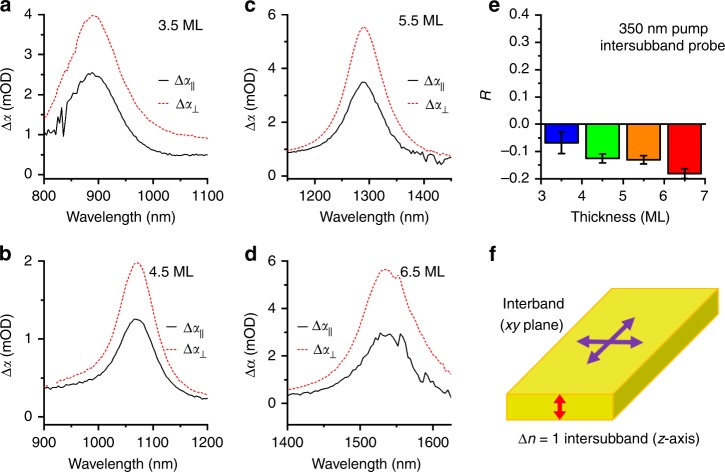
Polarization of intersubband absorption. NIR polarized transient absorption spectra for **a** 3.5, **b** 4.5, **c** 5.5, and **d** 6.5 ML CQWs. Samples were excited with horizontally polarized 350 nm light and probed with horizontal (parallel) and vertical (perpendicular) NIR white light probe beams. **e** Photoselection ansisotropy measured for 350 nm pump excitation and intersubband probe, calculated from the intensities of the perpendicular and parallel features. Error bars are calculated from the standard deviation of the anisotropy for delay times between 10 and 15 ps. **f** Schematic of the polarization properties of the interband excitations of CQWs and the observed intersubband transition reported (**a**)–(**d**)

As noted above, in epitaxial quantum wells, it is well known that the *E*_1_ → *E*_2_ intersubband transition is highly polarized in the short axis of the wells^18^. Photoselection anisotropy transient absorption can therefore confirm the *E*_1_ → *E*_2_ intersubband transition. Measurements of photoselection anisotropy using transient absorption are similar to fluorescence anisotropy measurements, except that in transient absorption, both excitation and probe dipoles relate to the absorption. A polarized excitation source (350 nm laser) photoselects a subset of the ensemble for which the absorption dipole is aligned with the excitation, then the sample is examined with parallel or perpendicular polarized probe beams. Polarization-resolved transient absorption spectra for the NIR features at a delay of 10 ps of CQWs are shown in Fig. 3a–d. In each case, the induced absorption feature is stronger in the polarization perpendicular to the pump beam. An isotropic sample of lead selenide quantum dots in Supplementary Fig. 4 confirms that the experimental setup is not the origin of the observed polarization effects. Unlike molecular systems, large CQWs do not undergo rotational depolarization on the measurement time-scale (Supplementary Fig. 5).

The intensity ratio of the photoinduced absorptions (or visible bleaches) is determined by the axes of the CQWs on which different excitations occur. The measured transient absorption anisotropy of an isotropic ensemble is

2 $$R = \frac{{\Delta \alpha _\parallel - \Delta \alpha _ \bot }}{{\Delta \alpha _\parallel + 2\Delta \alpha _ \bot }} = \frac{2}{5}r_{{\mathrm{pump}}}r_{{\mathrm{probe}}},$$

in which *r*_*i*_ is the anisotropy of the pump and probe wavelength intensity (*I*)^44^. For arbitrary isotropic ensembles, the range of anisotropy is from −0.2 to 0.4; assuming, as is reasonable in this case, that the two-dimensional plate of the CQW has degenerate long axes (*x*, *y*), *r*_*i*_ is $I_{zi} - I_{xi,yi}/I_{zi} + 2I_{xi,yi}$ with respect to the fluorophore axes, where the intensities may be normalized such that $I_x + I_y + I_z = 1$. When pump and probe absorption dipoles are exclusively in-plane ($I_{x} = I_{y} = 0.5$), the anisotropy is 0.1, which is indeed close to the anisotropy measured for the visible bleach features of the CQWs (Supplementary Fig. 6). This is also consistent with many previous works showing that interband transitions of CQWs are in-plane^5,6,45,46^. Non-idealities, like rectangular in-plane shape or bending and twisting of the CQWs—more prominent in thinner CQWs^47^—may impose some deviations from this idealized picture. Returning to Fig. 3e, the anisotropy values measured at the intersubband absorption are negative, with values reaching as low as −0.18. These results may only be achieved when the pump absorption dipole is polarized almost exclusively in-plane, but the probe absorption dipole is polarized almost exclusively in the unique out-of-plane axis as shown in the cartoon in Fig. 3f. Given that this is precisely what is observed in epitaxial quantum wells for the *E*_1_→*E*_2_ transition, polarized measurements support the assignment of the NIR-induced absorption features to the *E*_1_→*E*_2_ intersubband transition of the CQWs.

### Temperature-dependent intersubband absorption

**Fig. 4:**
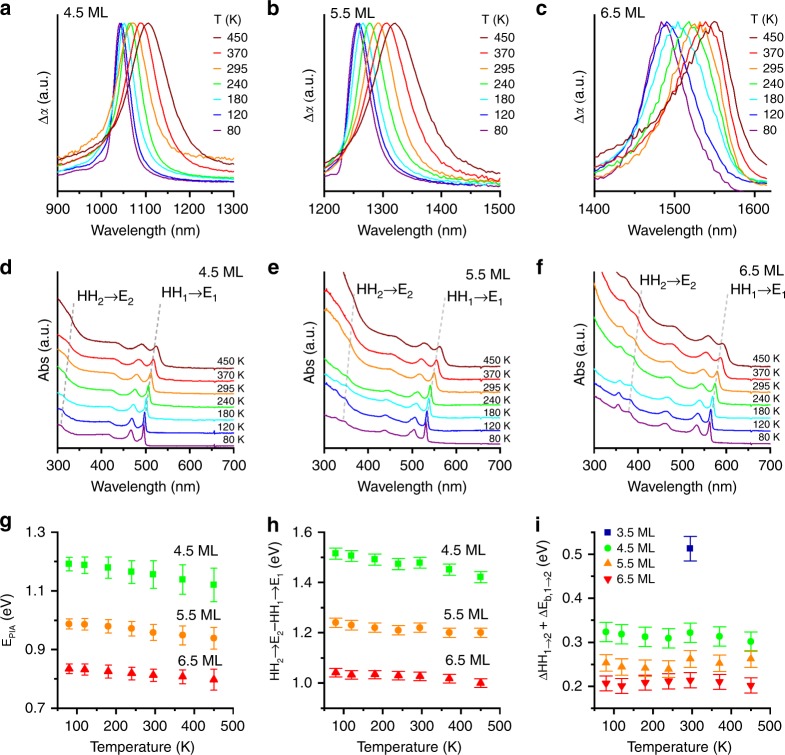
Temperature dependence of intersubband absorptions. NIR-photoinduced absorption of **a** 4.5 ML, **b** 5.5 ML, and **c** 6.5 ML CdSe CQWs. Transient absorption spectra are for 10 ps delay after 420 nm excitation at indicated temperatures. Static absorption spectra of **d** 4.5 ML, **e** 5.5 ML, and **f** 6.5 ML CdSe CQWs. **g** Fitted energies of the NIR-photoinduced absorption feature plotted vs. temperature. The bars represent the full-width at half-maximum of the feature. **h** ${E}\left[ {{\mathrm{HH}}_2 \to {E}_2} \right] - {E}\left[ {{\mathrm{HH}}_1 \to {{E}}_1} \right]$ from static absorption spectra of CdSe CQWs, plotted as a function of temperature. Error bars represent the summed error of the two band positions, based on the bandwidth of the measurement. **i** Energy gap of the observed intersubband absorptions in (**g**) with ${E}\left[ {{\mathrm{HH}}_2 \to {E}_2} \right] - {E}\left[ {{\mathrm{HH}}_1 \to {E}_1} \right]$ from (**h**). This gap is equivalent to the heavy-hole intersubband absorption energy and differences in the exciton binding energy of the *n* = 1 and *n* = 2 interband absorptions, which is printed on the plot. For reference, the 3.5 ML CQW data from room temperature is also included. Error is estimated as in (**h**)

The samples were additionally examined by temperature-dependent spectroscopy to ascertain the change in bandwidth and energy of the intersubband absorption features. Figure 4a–c show temperature-dependent photoinduced absorption, and with corresponding static absorption spectra of 4.5, 5.5, and 6.5 ML samples in Fig. 4d–f. Both intersubband and interband transitions red-shift at higher temperatures due to lattice expansion. Figure 4g plots the energy and bandwidth of the NIR intersubband absorption features as a function of temperature for the 4.5, 5.5, and 6.5 ML samples. Over the studied temperature range, the intersubband absorptions shift linearly with a thermochromic coefficient of −1.9 × 10^−4^, −1.4 × 10^−4^, and −9.9 × 10^−5^ eV/K for 4.5, 5.5, and 6.5 ML CQWs, respectively, which are somewhat smaller than reported values of the Varshni α term for the temperature-dependent band gap change of CdSe nanocrystals and CQWs^48–55^.

The bandwidth of the intersubband absorption feature (like interband absorptions) is temperature-sensitive due to the increased electron–phonon coupling that also influences the ensemble linewidths of the band-edge transitions^2,52,56^. For example, the 6.5 ML CQW intersubband absorption narrows from 70 to 34 meV full-width at half-maximum at 450 and 80 K, respectively. A pronounced asymmetry for the 6.5 ML CQW sample at high temperature is most likely due to poor linearity of the InGaAs array detector response for wavelengths >1575 nm. By comparison, intersubband absorption features in GaN/AlN epitaxial quantum wells of comparable energy (corresponding to c. 1.55 μm) show full-widths at half-maximum energies of 160 meV or greater^20,23^. Although intraband absorptions in zero-dimensional quantum dots can be similarly narrow, they typically have energies <500 meV. Interband transitions of mercury chalcogenide CQWs show line width of the absorption and photoluminescence features of 60 meV at room temperature at close to 900 nm^57^, which is narrower than the 3.5 ML CdSe CQW intersubband absorption and comparable to 6.5 ML CQWs under photoexcitation; however, the energy range of these interband transitions is presently limited.

Figure 4h displays the empirical upper energy limit ($E\left[ {{\mathrm{HH}}_2 \to E_2} \right] - E\left[ {{\mathrm{HH}}_1 \to E_1} \right]$) of the *E*_1_ → *E*_2_ intersubband transition at many temperatures, calculated from the optical absorption spectra. $E\left[ {{\mathrm{HH}}_2 \to E_2} \right] - E\left[ {{\mathrm{HH}}_1 \to E_1} \right]$ exceeds the observed intersubband transition energy for all thicknesses of CQW and all temperatures (Fig. 2b), due to hole intersubband transitions and exciton binding energy differences. Indeed, this energy gap is nearly constant with temperature for a given thickness of CQW, as demonstrated in Fig. 4i.

### Fluence-dependent effects on intersubband absorptions

**Fig. 5:**
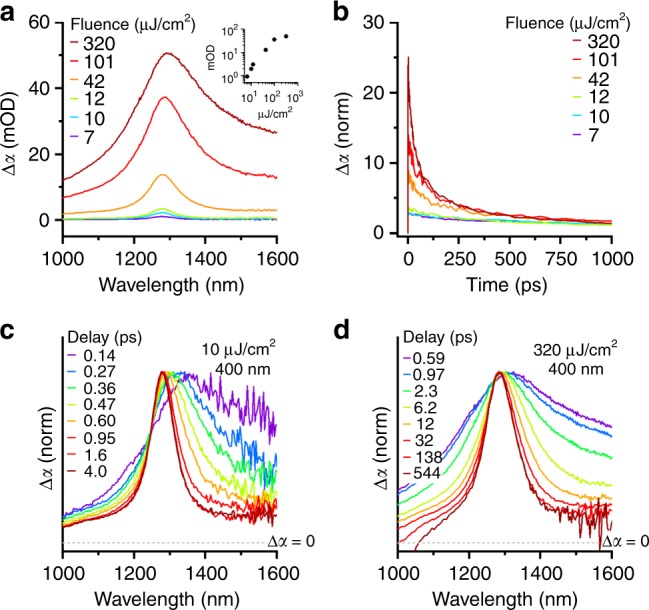
Auger and hot-carrier effects on intersubband absorption. **a** Transient absorption spectra of 5.5 ML CQW pumped at 400 nm at 2 ps delay for several indicated fluences. The inset data show the peak change in optical density of the photoinduced absorption vs. fluence. **b** Dynamics of 5.5 ML CQW-induced absorption feature at several fluences. Data are normalized by the average signal between 1500 and 2000 ps. **c**, **d** Transient absorption spectra of a 5.5 ML CQW sample pumped with 400 nm photons at **c** 10 μJ/cm^2^ and (**d**) 320 μJ/cm^2^ for several indicated delay times

Similar to excitonic absorptions of semiconductor nanocrystals, the intersubband absorptions of the CQWs display fluence-dependent Auger and hot-carrier effects. With higher pump fluences at early delay times, the strength of intersubband absorption increases (shown in Fig. 5a for a 5.5 ML CQW sample), but sub-linear growth is observed at the highest examined fluence as the CQW exciton transition saturates. Elevated pump fluences also initiate multiexciton physics, chiefly Auger recombination, which dramatically changes the kinetics of the intersubband induced absorption. Similar to other semiconductor nanomaterials, Auger recombination manifests as an accelerated decay of the Δ*α* signal, as shown in Fig. 5b, above the fluence threshold (here >10 μJ/cm^2^) that generates more than one electron–hole pair per CQW^58,59^. For the 5.5 ML CQW sample studied in Fig. 5, the biexcitonic Auger time estimated from fluence-dependent kinetics of the intersubband photoinduced absorption is 225 ± 50 ps, which is comparable to previous measurements based on time-resolved photoluminescence^9^.

Hot-carrier effects on the intersubband absorption features are chiefly dictated by the presence or absence of multiexciton Auger recombination, displaying distinct behavior based on the incident fluence. Fluence-dependent hot-carrier effects are well known in bulk materials^60^, epitaxial quantum wells^61^, and even CQWs^35^, but explicit spectroscopic investigations of the influence of hot carriers on intersubband resonances in epitaxial quantum wells are limited to demonstrations of line broadening^62^. At low fluences, as in Fig. 5c, the signature of hot-carrier absorption is the broadened and red-shifted photoinduced absorption, which decays within <2 ps to the spectrum observed at long delay times. Unlike electronic bleaches or photoluminescence, for which hot-carrier effects are manifest from blue-shifted spectral signatures at the band edge, the intersubband absorption predominantly shows a red-shift because the energy difference between the *E*_1_ and *E*_2_ bands effectively decreases for hot carriers in the *E*_1_ band. The specific nature of intersubband transition broadening for hot carriers also depends on the non-parabolicity of the bands. Under high fluence conditions, as shown in Fig. 5d, thermal relaxation of carriers observed via the intersubband absorption is spectrally apparent from the broadened response for at least 100 ps after the excitation pulse, chiefly arising from continuous heating of electrons due to Auger recombination, although lattice heating may also play a role on such time-scales^35^.

### Optical switching with intersubband transitions in CQWs

**Fig. 6:**
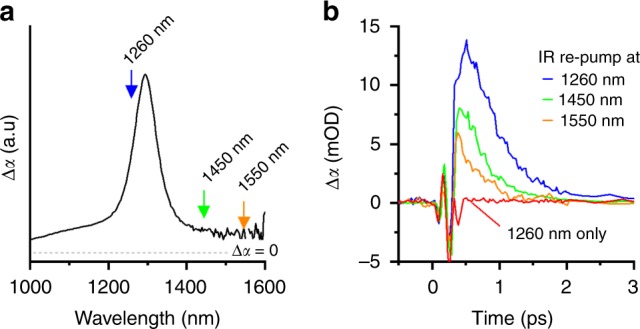
Two-color pumping of colloidal quantum wells. **a** NIR-photoinduced absorption of a 5.5 ML CQW ensemble following 400 nm excitation at 50 ps delay. The gray dashed line indicates the position of zero change in absorption. **b** Transient dynamics probed at 560 nm) using an unchopped 400 nm excitation followed, at 50 ps, by chopped IR pump pulses of various indicated energies. The observed dynamics are those induced by the chopped IR pump pulses, which re-pump the already excited CQWs. The full-width at half-maximum of the ~30 fs pump pulses is ~100 meV. Excitation at 1260 nm in the absence of the unchopped 400 nm excitation (shown in red) yields only a transient Stark shift

Intersubband transitions in epitaxial quantum wells have long been viewed as promising candidates for optical switching technologies^21,63,64^, in addition to their extensive use in quantum cascade lasers and IR detectors. Primarily, this is because excitation of carriers within subbands typically results in sub-picosecond relaxation, particularly compared with interband processes (e.g., up to 50 ns in 900 nm band-edge HgTe CQWs^57^) or even intraband relaxation in quantum dots (e.g., many picoseconds in CdSe quantum dots^65^). Whereas optical excitation can produce an add/drop switch with appearance and decay of the NIR absorption feature, to create a faster optical switch using intersubband absorptions of CdSe CQWs, which have no static intersubband absorption in their as-synthesized state, two pump excitations are used. First, an unchopped above-band-gap blue excitation (400 nm) is used to excite the sample and generate an intersubband absorption resonance shown in Fig. 6a. The second, chopped, IR pump pulse is delivered at a delay of 50 ps (after cooling of carriers), with the center wavelength of such pulses tuned near the intersubband absorption feature. Transient spectra were collected in the visible near the band-edge absorption features by comparing the IR pump on vs. IR pump off signals. The fluence of the IR pump pulse was maintained sufficiently low for all wavelengths (c. 500 μJ/cm^2^) that multiphoton absorption and concomitant state filling was not observed experimentally, as shown in Fig. 6b. The transient signal near the band-edge HH to E_1_ transition represents the dynamics of heating and cooling of electrons in the conduction band of CQWs.

The IR re-pump pulse closest to the intersubband absorption resonance generated the largest induced absorption feature at red side of the CQW band-edge absorption feature. However, off-resonance wavelengths also result in transient induced absorption, most likely by exciting the weaker, but continuous photoinduced absorption arising from intraband transitions of photoexcited carriers. The positive sign of the Δ*α* signal (induced absorption) is expected on the red edge of the band-edge absorption feature: exciting carriers in the *E*_1_ band of the CQWs transiently heats those carriers above ambient temperature, reducing their occupation of the *E*_1_ band edge and permitting increased absorption until carriers cool back to ambient temperature. Cooling back to room temperature occurs with an exponential lifetime of 530 fs, determined by fitting the decay kinetic with 1260 nm IR re-pump (see Supplementary Fig. 7).

## Discussion

CdSe CQWs display photoinduced or charging-induced absorption features at wavelengths spanning 1550 nm for 6.5-ML-thick CQWs to 900 nm for 3.5-ML-thick CQWs at room temperature. A combination of detailed analysis of static optical spectra, dynamics, and polarized spectroscopy confirms that the observed transitions are intersubband transitions of electrons from the first electronic shelf to the second electronic shelf of the CQWs. This observation maps well on to the band structure models developed for epitaxial quantum wells, as has already been found for interband transitions, but with the distinct advantages emergent from colloidal synthesis, such as scalability and lifting of epitaxial constraints. Recent synthetic work to expand the range of CQWs available to thinner and thicker sizes^32–34^ may further expand the range of intersubband transitions into the MIR and visible. The narrow spectral bandwidth of the intersubband absorptions in CQWs compared to epitaxial systems is particularly noteworthy. Recent work showing the critical role of surface termination on the lattice strain of CQWs, CQW band gap, and CQW transition line widths underlines the potential of colloidal systems to relax to a state with reduced bandwidth compared to epitaxial systems^66–68^. In the future, this can be explicitly explored with atomically precise epitaxial interfaces.

These results convey that many related systems, which have similar electronic structure, such as CQWs of other compositions, two-dimensional van der Waals quantum wells, or quasi-two-dimensional perovskite materials, are also likely to show similar intersubband absorptions. Previous investigations of these van der Waals structures, in particular, have already demonstrated intersubband transitions in the mid-IR^30^. Variation of composition to achieve larger band gaps, which are typically accompanied by larger subband spacings, and greater asymmetry of carrier effective masses are likely to drive such intersubband transitions to higher energy. Controlled doping and self-assembly of CQWs may enable NIR technologies analogous to those of epitaxial quantum wells, such as detectors and multiwell light-emitting technologies.

## Methods

### Materials

Cadmium acetate hydrate (≥99.99%, Aldrich), cadmium chloride (99.99%, Aldrich), hexanes (99.9%, Fisher), methylcyclohexane (≥99%, anhydrous, Aldrich), selenium (99.99%, powder, ~100 mesh, Aldrich), 1-octadecene (90%, technical grade, Aldrich), oleic acid (90%, technical grade, Aldrich), cadmium nitrate (97%, Aldrich), tetrabutylammonium perchlorate (>99.0%), benzoic acid (99.5%, Aldrich), and sodium myristate (>98%, Aldrich) were sourced from commercial suppliers and used as received. Solvents used were sourced from commercial suppliers and were ACS grade or higher.

### Synthesis

Synthesis of CQWs followed literature procedures^1,2,9,32^. Cadmium myristate was synthesized by co-dissolving sodium myristate and cadmium nitrate in methanol and washing the white solid with methanol and acetone. CQWs (3.5 ML) were synthesized by mixing 240 mg of cadmium acetate, 150 μL oleic acid, and 15 mL of octadecene. The mixture was held under vacuum for 1 h at 80 °C, and then heated under nitrogen to 180 °C, whereupon a 150 μL solution of 1 M selenium dissolved in trioctylphosphine was rapidly injected. The reaction was held at 180 °C under nitrogen for 10 min, and then cooled to room temperature and precipitated with isopropanol and re-dispersed in methylcyclohexane. CQWs (4.5 ML) were prepared with 12 mg selenium powder, 170 mg cadmium myristate, 15 mL octadecene, which are held under vacuum at room temperature for 1 h, and then heated under nitrogen to 240 °C. At 195 °C, under nitrogen counterflow, 40 mg of finely ground cadmium acetate was added rapidly. The reaction was held at 240 °C for 10 min, and then cooled. After the reaction was completed, the heating mantle was removed and 2 mL of oleic acid was injected followed, at ~100 °C, by 10 mL of toluene. NPLs were precipitated from the reaction medium by centrifugation at 15,000 r.p.m. and re-dispersed in methylcyclohexane. Synthesis of 5.5 ML CWSs was achieved by mixing 170 mg cadmium myristate in 14 mL octadecene, holding under vacuum for 1 h at room temperature, and then heated under nitrogen to 250 °C. At 250 °C, a dispersion of 12 mg selenium in 1 ML octadecene (dispersed with sonication and vigorous mixing) was rapidly injected, followed, after 1 min, by the addition of 90 mg cadmium myristate with nitrogen counterflow. The reaction proceeded for an additional 15 min. After removal of the heating mantle, 2 mL of oleic acid was injected followed, at ~100 °C, by 10 mL of toluene before being cooled to room temperature. Samples were isolated by centrifugation of the reaction mixture at 15,000 r.p.m., with the sample re-dispersed in methylcyclohexane. CQWs (6.5 ML) were prepared by mixing 170 mg cadmium myristate and 14 mL octadecene, held under vacuum for 30 min at 85 °C, and then heating under nitrogen to 250 °C. At 250 °C, a dispersion of 12 mg selenium in 1 ML octadecene (dispersed with sonication and vigorous mixing) was rapidly injected, followed, after 20 s, by the addition of 60 mg cadmium myristate with nitrogen counterflow. The reaction proceeded for an additional 60 s, and then a dropwise injection of 0.15 mL of 0.5 M cadmium chloride in water over 2 min. After an additional 3 min, the reaction was cooled by removing the heating mantle. At 150 °C, 2 mL oleic acid and 15 mL methylcyclohexane was injected. The reaction medium was centrifuged to precipitate a mixture, which contains 6.5 ML CQWs, thinner CQWs, and other small particles, which were separated by size-selective precipitation of methylcyclohexane dispersions.

### Steady-state spectroscopy

Room temperature static absorption spectra of methylcyclohexane solutions were collected using a Cary 50 UV–Vis spectrometer. Temperature-dependent measurements were made on solid films drop-cast from methylcyclohexane on to sapphire windows using a deuterium halogen lamp focused through the samples mounted on sapphire in a nitrogen-cooled cryostat and detected using an Ocean Optics spectrometer. Room temperature solution NIR measurements were performed using a Nicolet 6700 FT-IR (Fourier transform infrared) with an InGaAs detector with samples dispersed in carbon tetrachloride in quartz cuvettes.

### Microscopy

Transmission electron microscopy images of CQW samples were collected using a JEOL 2100F microscope operated at 200 keV.

### Time-resolved spectroscopy

Transient absorption data was collected using Ultrafast Systems equipment operating at either 5 kHz (~100 fs pulses) or 2 kHz probe frequency (~35 fs pulses), with alternate pump shots blocked with a mechanical chopper. Methylcyclohexane dispersions in 1 mm quartz cuvettes with a band-edge optical density of ~0.5 were used for experiments at room temperature, although the response of solid films on glass or sapphire were not substantially different. For temperature-dependent measurements, solid films were prepared by drop-casting methylcyclohexane dispersions on to a sapphire window to achieve a similar optical density. For polarization-sensitive measurements, a linear polarizer was placed after the sample to select parallel and perpendicular components of the white probe light. The strength of different polarizations of the probe light was adjusted to achieve similar intensities with both polarizations by adjusting the relative angle of the sapphire crystal used to generate the white light compared to the driving 800 nm pulse. For two-pump pulse measurements, the experiment was first configured to obtain mutually overlapping spots of the NIR and blue (400 nm) pump beams and the probe beam, with the two pump beams separated by ~50 ps in time. Overlap of the NIR beam was initially performed at high fluence to induce multiphoton absorption. (Multiphoton absorption can also induce the same intersubband absorptions.) To isolate dynamics associated with the IR pump pulse on a photoexcited sample, the 400 nm pump beam was unchopped (2 kHz) and the IR pump pulse was chopped at 1 kHz.

### Spectroelectrochemistry

Spectroelectrochemistry was performed similar to an earlier work^69^. Samples were prepared by drop-casting CQWs dispersed in methylcyclohexane (optical density at band-edge exciton >3 cm^−1^) on to gold interdigitated electrodes designed for a home-built spectroelectrochemical cell. Thin films were ~200 nm thick, estimated from optical absorption. Measurements were performed using a Nicolet S50 FT-IR. The drop-cast thin films were treated in air with a 5 mg mL^−1^ benzoic acid solution in methanol for 1 min, washed in air by immersion in clean methanol solutions three times for 30 s, and then blown dry with nitrogen. The film coverage of the electrodes varied substantially after this processing due to delamination during the washing steps. The electrolyte was 0.1 M tetrabutylammonium perchlorate in dried and degassed propylene carbonate and the sample cell was loaded in a nitrogen glovebox to minimize oxygen contamination For each measurement at a new voltage, a background at −0.044 vs. saturated calomel electrode was first collected and the current measured between the interdigitated electrodes was allowed to stabilize (typically within 3 min) before starting the FT-IR measurement. Using the interdigitated working electrodes, conductance of the channel was also measured at various applied potentials. Potentials of data presented in the work were chosen to achieve adequate signal to noise and do not reflect the minimum sufficient voltages of band filling for the samples.

## Supplementary information

Supplementary Information

## Data Availability

All source data are available from the authors.
