# Supplementary material for: Polarized near-infrared intersubband absorptions in CdSe colloidal quantum wells
Source: Nat Commun. 2019 Oct 4;10:4511. doi: 10.1038/s41467-019-12503-z (PMC6778118; doi:10.1038/s41467-019-12503-z)
Supplement: Supplementary file 1 — Supplementary Information [file 41467_2019_12503_MOESM1_ESM.pdf]

Supplementary Information

Polarized Near-Infrared Intersubband Absorption in CdSe Colloidal Quantum Wells

Diroll et al.

Supplementary Discussion

Effective Mass Approximation modeling. In order to estimate the positions of the hole and electron energy levels in CdSe colloidal quantum wells (CQWs) we used the approach described by Efros et al.,[1] although updated values were used based upon present understanding of the CQW thicknesses. Briefly, the position of the bulk bands is calculated within the formalism of the eight-band Pidgeon-Brown model. The bulk material parameters used in the calculation are summarized in table 1. The energy of the quantum sized level was then found by scaling the band energy by the canonical energy relationship in quantum wells:

$$E_k(n) = \frac{\hbar^2\pi^2 n^2}{2mL_z^2} \tag{1}$$

where m is the free electron mass and L_z is the thickness of the well and n is the principal quantum number of the envelope wave function. By solving the coupled equations of the Pidgeon-Brown Hamiltonian with the boundary conditions of an infinitely high potential barrier, one obtains the positions of the conduction band (E_n), the heavy hole band (HH_n), the light hole band (LH_n), and the split off hole band (SO_n). The thickness (L_z) of the nanoplatelet was taken as:

$$L_z = \frac{wa_0}{2} \tag{2}$$

where w is the number of monolayers using the same convention used in the text (i.e. 3.5, 4.5, 5.5, or 6.5) and a_0 is the bulk lattice constant of zb-CdSe. Since the approximation of an infinitely high barrier results in an artificially high confinement energy we extended L by an additional 0.32 nm as a global empirical correction using the expression below:

$$L_{z,eff} = \frac{wa_0}{2} + 0.32\text{nm} \tag{3}$$

This correction was chosen to make the calculated heavy hole optical transition energy match the experimentally observed value for 6.5 ML platelets as described previously.[2] In practice, the EMA approach reproduces the qualitative trends observed in intersubband and interband absorption properties but overestimates the energy of transitions, particularly those at higher energy and thinner CQW size.

Supplementary Table 1. Constant values used in EMA model.

Constant	Value
a	0.608
E_g (eV)	1.66
E_p (eV)	16.5
Δ (eV)	0.39
α	-1.54
γ_1	-0.18
γ_2	-0.65

Derivation of intersubband transition energy from optical absorption spectrum of colloidal quantum wells. The energy of each experimentally-observable interband transition of heavy hole states $E[HH_n \rightarrow E_n]$ is adjusted by the exciton binding energy according to $E[HH_n \rightarrow E_n] = (E_n - HH_n) - E_b[HH_n \rightarrow E_n]$. (Note that the energy difference between E_n and HH_n is not directly observable due to the exciton binding term, whereas experimentally-observable transition energies are indicated with an arrow.) As the interband transitions with *Δn* = 0 are observed, these may be used to estimate expected intersubband transition energies according to according to

$$\begin{aligned} E[HH_n \rightarrow E_n] - E[HH_m \rightarrow E_m] \\ = E[E_m \rightarrow E_n] + E[HH_m \rightarrow HH_n] - E_\mathrm{b}[HH_n \rightarrow E_n] + E_\mathrm{b}[HH_m \rightarrow E_m] \end{aligned} \quad (4)$$

in which $E[E_m \rightarrow E_n]$ and $E[HH_m \rightarrow HH_n]$ may be recognized as the intersubband absorption transition energies of the electron and heavy hole, respectively. The above equation is re-arranged in the main text to arrive at the energy $E[E_1 \rightarrow E_2]$ of the $E_1 \rightarrow E_2$ transition exploiting the experimentally observable transitions $HH_2 \rightarrow E_2$ and $HH_1 \rightarrow E_1$. Due to substantial absorption from surface-bound organic ligands at ultraviolet energies and broadening of the optical spectral features, definition of higher (n > 2) states is not achievable in all samples studied.

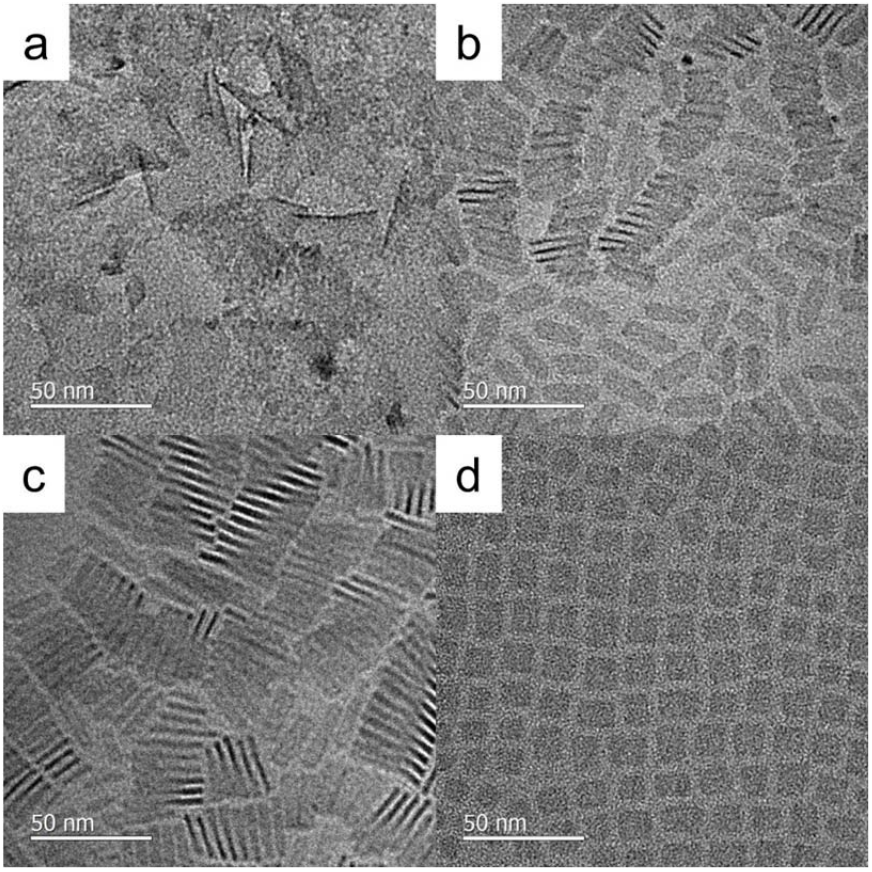

Supplementary Figure 1. Microscopy of Colloidal Quantum Well Samples. Transmission electron microscopy images of (a) 56.1±14.1 nm × 34.8±7.1 nm rectangular cross-section 3.5 ML CQWs, (b) 18.2±1.9 nm× 12.3±1.3 nm rectangular cross-section 4.5 ML CQWs, (c) 26.0±3.4 nm × 7.6±1.2 nm rectangular cross-section 5.5 ML CQWs, and (d) 9.4±1.3 nm square

cross-section 6.5 ML CdSe CQWs. The samples display negligible confinement in the lateral directions. Examination of other samples of the same thickness did not at any point result in substantially different optical properties.

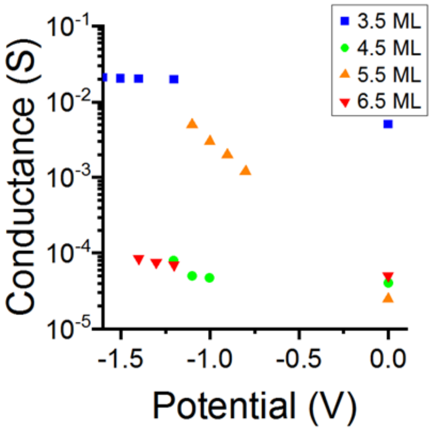

Supplementary Figure 2. Conductance of Colloidal Quantum Well Films under Bias. Measured conductance for spectroelectrochemistry measurements for films of CQWs with different thicknesses.

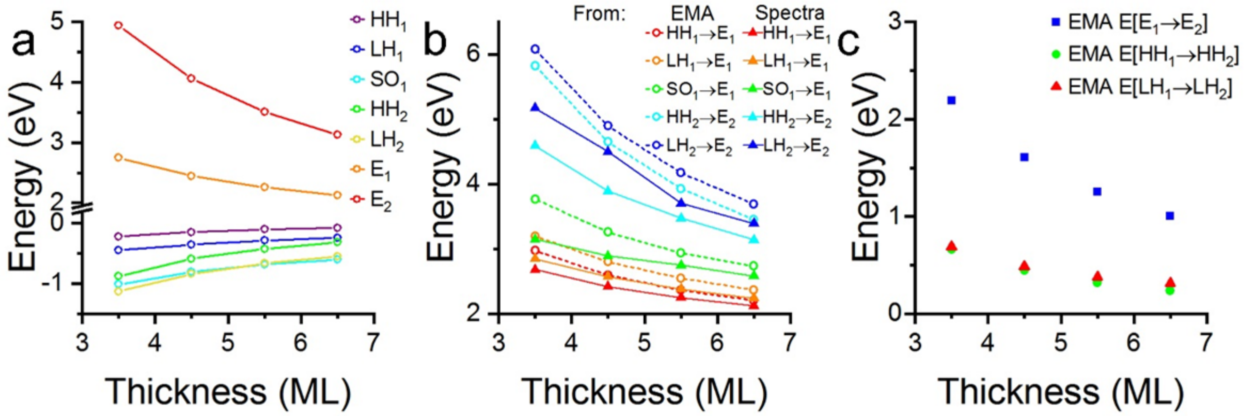

Supplementary Figure 3. Effective Mass Modeling of Colloidal Quantum Wells. (a) Energy levels of hole and electron states of the n = 1 and n = 2 quantum levels, calculated according to an EMA model. (b) Comparison of the transition energy of interband absorption resonances calculated by an EMA model, shown in open circles, and determined experimentally, in closed triangles. (c) Estimated energies of the first intersubband transition of electrons versus CQW thickness and the heavy- and light-hole transitions.

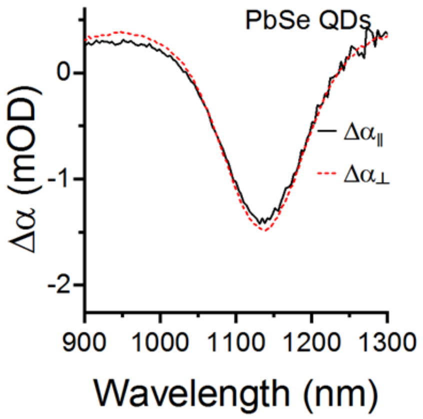

Supplementary Figure 4. Polarization Test of Non-Polarized Absorber. Transient absorption anisotropy spectra at 10 ps delay for ~3.5 nm PbSe quantum dots, with 350 nm polarized excitation. Estimated anisotropy of the sample, presumed to derive from the experimental apparatus, was -0.015±0.007. This is comparable to the standard deviation of anisotropy the estimates for the CQWs.

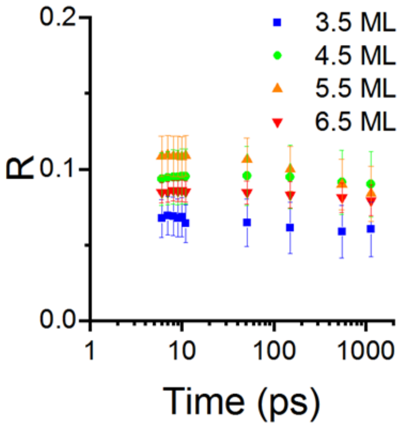

Supplementary Figure 5. Dynamics of Photoselection Anisotropy of Colloidal Quantum Wells. Visible transient absorption anisotropy of the heavy-hole bleach feature for CQWs of various thicknesses. The samples were pumped with a polarized 350 nm laser.

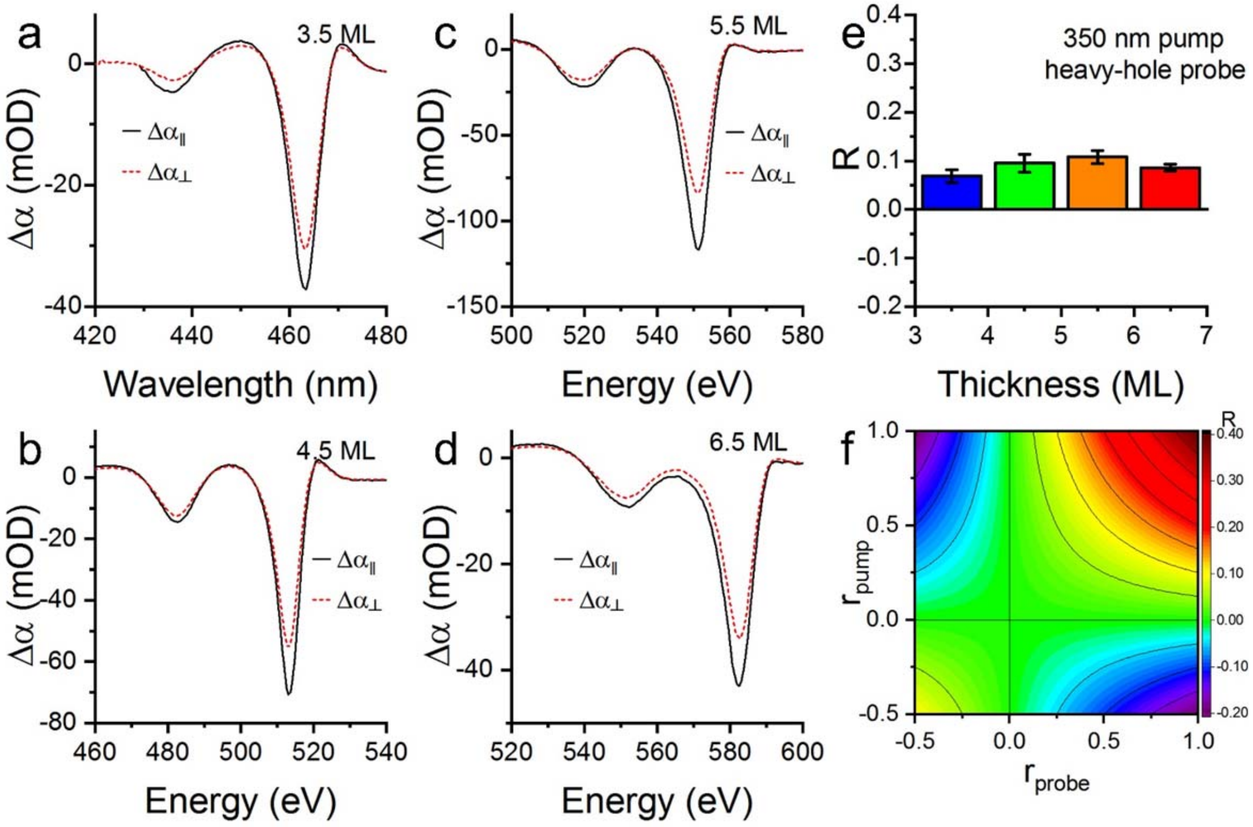

Supplementary Figure 6. Visible polarization properties of Colloidal Quantum Wells. Polarized transient absorption spectra at 10 ps delay for (a) 3.5, (b) 4.5, (c) 5.5, and (d) 6.5 ML CdSe CQWs used in this study. (e) Measured photoselection anisotropy at the heavy-hole band of each sample with 350 nm pump excitation. Error bars are estimated from the standard deviation in anistotropy values estimated from 5-10 ps delay. (f) Contour map of the possible values of anisotropy (R) for a fluorophore with two degenerate axes as described in the text. The polarization properties of the interband absorptions occur in the third quadrant; polarization properties of the intersubband absorptions are in the fourth quadrant. Derivation of this relation can be found in the literature.[3]

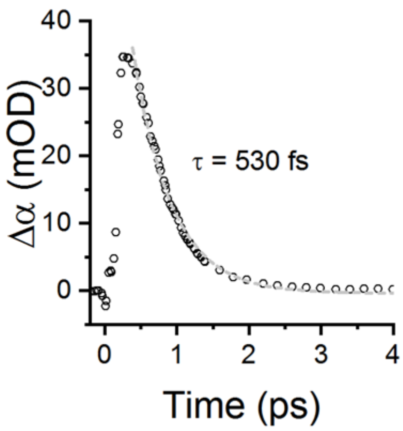

Supplementary Figure 7. Two-Color Pump Dynamics. Decay of photoinduced absorption in two pump experiments, measured at 560 nm, with an exponential decay fitted with the gray line.

Supplementary References

1. Ithurria, S. *et al.* Colloidal nanoplatelets with two-dimensional electronic structure. *Nat. Mater.* **10,** 936–941 (2011).

2. Cho, W. *et al.* Direct Synthesis of Six-Monolayer (1.9 nm) Thick Zinc-Blende CdSe Nanoplatelets Emitting at 585 nm. *Chem. Mater.* **30,** 6957–6960 (2018).

3. Diroll, B. T. *et al.* Interpreting the Energy-Dependent Anisotropy of Colloidal Nanorods Using Ensemble and Single-Particle Spectroscopy. *J. Phys. Chem. C* **117,** 23928–23937 (2013).
